# Alcohol Use and Operational Readiness in the U.S. Active-duty Military: A Review of Burden, Culture, and Barriers to Care

**DOI:** 10.1007/s11920-026-01700-5

**Published:** 2026-07-18

**Authors:** Rebecca Lobach, Rohul Amin, Sarah Maggio, Kathleen Huber

**Affiliations:** 1 Chapel Hill, North Carolina USA; 2 Department of Psychiatry and Medicine, USUHS, PO Box 88, Dayton, MD 21036 USA; 3 Department of Psychiatry, USUHS Henry M Jackson Foundation for the Advancement of Military Medicine, Inc., Bethesda, MD USA

**Keywords:** Military Personnel, Alcohol Use, Operational Readiness, Mental Health, Military Culture

## Abstract

**Purpose of Review:**

This review examines alcohol use among U.S. military personnel, including patterns of use across the full spectrum of consumption, including behaviors not meeting the Diagnostic and Statistical Manual of Mental Disorders (DSM-5) criteria for alcohol use disorder. It examines the prevalence and aspects of military life, culture, and context that may serve as important contributors to and risk factors for developing unhealthy, excessive, or disordered alcohol use. This review discusses efforts towards prevention, interventions, and policies pertaining to alcohol use in the military and identifies areas that would benefit from future research.

**Summary:**

Alcohol use remains a critical determinant of military health, operational readiness, and force lethality. While the Department of War (DoW) has invested in developing a modern framework for harm reduction, rates of binge and heavy drinking in the military continue to exceed that of civilians. Unique challenges of military life, including operational and institutional stressors, stigma and barriers to care, and factors of military social culture and context, coalesce to normalize and sustain unhealthy alcohol consumption within the force. Future efforts must focus on evaluating current interventions and tailoring evidence-based programs to address the needs of a diverse military population.

## Introduction

**Fig. 1:**
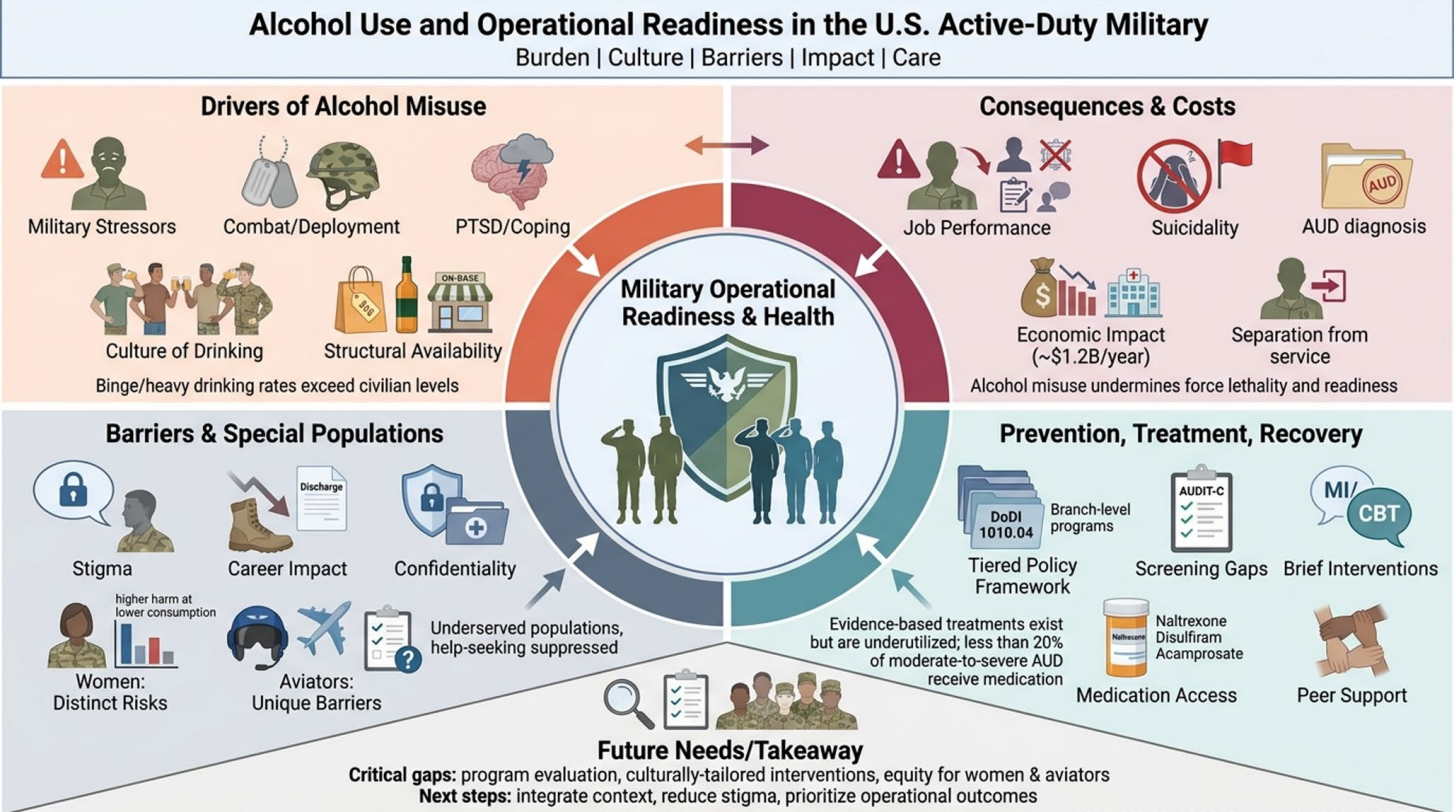
This conceptual summary figure maps the multifaceted relationship between alcohol use and U.S. active-duty military operational readiness, outlining key drivers, consequences, barriers to care, and current treatment frameworks. The figure represents the authors’ interpretation of the evidence presented in this review. The figure was created with assistance from figurelabs.ai tool based on the synthesis of evidence presented in this review. The authors reviewed the output and take full responsibility for its content

Alcohol use in the military should be understood within the framework of interaction between individual vulnerability, cultural reinforcement, and structural factors including policies. Historically, alcohol has played a complex role within the United States military both on and off the battlefield. Alcohol has been used as a medical tool [1–4] and as a mechanism for psychological management [5]. Even as medical science advanced and effective clinical alternatives emerged for wound care and pain management [6,7], the use of alcohol for its psychoactive effects persisted [8–12], often used for stress mediation, social bonding, and comradeship [5]. This review examines the prevalence and patterns of harmful drinking across the active-duty force, the cultural and structural factors that sustain it, and the current state of prevention, treatment, and recovery programming available to Service Members, with the aim of informing evidence-based solutions for the Warfighter (Fig. 1).

## Prevalence of Alcohol use in the Military

Evaluating the prevalence of alcohol use in military populations presents a unique set of challenges that differ from civilian epidemiological research. Influence from military culture and context, including mental health stigma, potential career repercussions, and complex leadership dynamics, can have a profound impact on care-seeking and self-reported drinking behaviors [9,13–15]. While military cultural factors may obscure the full scope of alcohol use, the Military Health System maintains tracking of formal diagnoses to monitor health trends across the services.

The Armed Forces Health Surveillance Division releases monthly data reports on illnesses, injuries, and health trends among U.S. military personnel. A recent report examining diagnoses of mental health disorders among the active component of the U.S. military indicated that there were 70,729 incident diagnoses of alcohol-related disorders between 2019 and 2023, accounting for 7.3% of all mental health diagnoses, and ranking among the top six most common incident mental health diagnoses for that reporting period [16]. Key incidence rates of alcohol-related disorders were notably higher among males aged 20–24 and those serving in combat-related occupations such as infantry, artillery, and combat engineering [16]. While clinical records provide a report of those who have entered the medical system, larger-scale surveys are necessary to capture the broader, often sub-clinical, patterns of alcohol consumption across the entire force.

The Health Related Behaviors Survey (HRBS) is the Department of War’s (DoW) flagship survey for evaluating the health, health-related behaviors, and well-being of Service Members, including data on the prevalence and patterns of alcohol use within the military [17]. To mitigate career concerns and encourage honest reporting, the survey is voluntary, confidential, and managed by a civilian third party that provides the DoW with only aggregated, de-identified data. The most recently published HRBS found that 34.0% of active component Service Members reported binge drinking, defined as consuming five or more drinks on the same occasion for men and four or more drinks for women, exceeding rates of adults in the general U.S. population (26.5%) ^18^. Heavy drinking, defined here as binge drinking at least one day each week in the past 30 days [18], was reported by 9.8% of Service Members. The highest rates for binge and heavy drinking were seen in the Marine Corps, while the Air Force had lower rates of binge and heavy drinking compared to other services [17]. Taken together, these data highlight the gap between clinical diagnoses and broader behavioral patterns, demonstrating that no single data source captures the full picture of alcohol use among military personnel.

The military alcohol literature also has a meaningful longitudinal infrastructure, anchored primarily in the Millennium Cohort Study, which has tracked drinking behaviors prospectively across up to 12 years and linked alcohol misuse to separation status, service component, and combat exposure [19, 20], enabling further examination of alcohol use patterns and insight into potential risk and protective factors across the military career lifecycle.

## Risk and Protective Factors for Harmful Alcohol use in the Military

Factors associated with harmful alcohol use can be individual, military-specific, or structural. Among military personnel, there is widespread perception that military culture is supportive of drinking [9, 17, 21], and there is a strong, positive correlation between positive perceptions of drinking culture in the military and alcohol-related outcomes such as excessive drinking, serious consequences, risky driving, and productivity loss [9, 22]. Normative beliefs have also played a role in alcohol consumption among military personnel, with individual drinking levels positively associated with overestimates of peer consumption [23], and accepted drinking norms surpassing those in the general population [24]. Longitudinal evidence further supports a causal interpretation, with evidence that normative beliefs about heavy drinking predicting changes in drinking behavior over time [25].

This culture is sustained by specific structural and institutional features including tax-free, discounted alcohol in stores on military installations [26–29], and inconsistent policy enforcement [5, 22, 26, 27]. The DoW has taken some steps to limit on-base alcohol availability, such as policies that prohibit late night sales of alcohol in exchange outlets on installations [30, 31]. This policy began in 2013 for the Navy and Marine Corps, but was not applied to the Army and Air Force until 2024 [30, 31]. These changes were made in response to recommendations from the DoW’s Suicide Prevention and Response Independent Review Committee in 2023 [32]. The committee also recommended banning the promotion of alcohol on DoW property and increasing on-base alcohol prices [32]. However, adoption and implementation of these additional measures remain unclear.

The unique demands of military life, such as combat exposure, deployment cycles, and operational tempo, create stressors for military personnel that are distinct from those encountered by civilians [33]. Deployment status alone is not a strong predictor of alcohol consumption [19, 20, 34], but specific characteristics of the deployment experience have been shown to increase risk for developing harmful drinking patterns [12, 35]. Deployment-related experiences including combat-related traumas, Post-Traumatic Stress Disorder (PTSD) symptoms, and other psychological distress have been associated with increased frequency of drinking [35, 36]. Beyond increased consumption, combat-exposed personnel are more likely to screen positive for Alcohol Use Disorder (AUD) [37], with exposure to life-threatening situations and atrocities conferring the greatest risk [38]. Additionally, the frequency and duration of deployment exposure show a dose-response relationship with drinking intensity [26, 39]. Moreover, the risk of sustained unhealthy drinking may persist beyond active service, with longitudinal evidence showing elevated odds of continued heavy and problem drinking among veterans and Reserve or Guard members compared to actively serving personnel [20]. Notably, personal life stressors experienced during deployment may also contribute to new-onset heavy drinking, independent of combat exposure [36].

The self-medication hypothesis, which postulates that individuals use alcohol to cope with trauma-related distress and mental health symptoms, is the predominant model for understanding PTSD-alcohol comorbidity in military populations [40, 41]. Meta-analytic evidence supports this pathway, with coping motives strongly mediating the relation between PTSD symptoms and harmful alcohol use [42]. Additionally, evidence shows that combat veterans are significantly more likely than other trauma-exposed men to endorse drinking to cope with PTSD symptoms [43]. Among Service Members, coping motives uniquely predict alcohol-related problems beyond drinking quantity alone [44].

Beyond individual coping motives, group-level factors also influence alcohol use. Unit cohesion encompasses the bonds of trust, shared purpose, and mutual support within military groups, and is widely recognized as protective against mental health problems. Higher cohesion is associated with reduced risk of PTSD, psychological distress, and suicidal ideation following combat deployment [45–49]. At the unit level, cohesion has been associated with fewer behavioral health problems and lower alcohol misuse in the Army National Guard Soldiers following deployment [49]. However, the relationship between cohesion and alcohol use is not straightforward. Among U.S. Marines, unit-level cohesion was positively associated with alcohol use while simultaneously predicting fewer disciplinary violations [50]. Research on Reservist or Guard Soldiers’ social networks found that having more peer-based social reinforcement and heavy-drinking ties was associated with increased alcohol problems, while for deployed Soldiers, larger military-affiliated social networks were actually protective [51]. Other studies have found no relationship between unit cohesion and alcohol consumption [52, 53]. Together, these findings suggest that cohesion’s effects are domain-specific and context-dependent. The same social bonds that reduce psychological distress may normalize heavy drinking, acting as a mechanism through which group identity is expressed and reinforced.

## The Impact of Alcohol use in the Military

Alcohol use exacts a heavy toll on the U.S. military across multiple domains and is associated with many operationally relevant adverse outcomes. At the individual level, negative outcomes follow a dose-response pattern, with heavy drinkers reporting nearly three times the rate of serious consequences and more than twice the rate of productivity loss compared to moderate drinkers [54]. Binge drinkers in the active-duty force show markedly elevated odds of job performance problems, alcohol-impaired driving, and criminal justice involvement [55]. Higher levels of alcohol consumption in military personnel have also been shown to correlate with increased likelihood of having considered or attempted suicide, and drinking to fit in or avoid rejection has shown to be associated with suicidality [56]. Alcohol use disorder has also been associated with a higher rate of separation from service [57, 58]. Administrative record studies have further established that AUD diagnoses predict negative military separation and elevated healthcare utilization within one to two years of post-deployment diagnosis [59].

At the organizational level, alcohol-related emergency department and inpatient encounters occurred at a rate of 75.3 per 10,000 person-years between 2009 and 2018, with over 17% involving co-occurring injuries, most commonly intentional in nature [60]. The economic burden of alcohol use on the DoW is substantial. In the most comprehensive cost analysis to date, it was estimated that alcohol use among TRICARE Prime beneficiaries cost the DoW $1.2 billion annually in 2006, of which roughly two-thirds was attributable to reduced readiness and misconduct, and the remainder to excess medical costs [61]. Although TRICARE Prime beneficiaries include active-duty members, retirees, and dependents, the readiness and misconduct costs ($745 million) are attributable primarily to the active-duty force, while excess medical costs ($425 million) are distributed across all beneficiary categories [61]. Beyond direct costs, alcohol use drives productivity losses through increased lateness, absenteeism, reduced performance, and on-the-job injuries among heavy-drinking personnel [9, 62]. Economic modeling suggests that interventions such as comprehensive screening with brief intervention, a 20% price increase on alcohol sold at military installations, or web-based education programs could each yield $75–129 million in annual savings [61], and a brief alcohol misconduct prevention program implemented at one Air Force training site returned $4–6 for every dollar invested [63]. Collectively, these findings underscore that alcohol use is not merely a personal health concern, but a force readiness problem with measurable costs that warrant sustained investment in evidence-based prevention and intervention.

## Alcohol Prevention, Treatment, and Recovery Programs in the Military

### Policy Framework and Overarching Structure

The DoW’s approach to problematic alcohol use is governed by a tiered policy structure that establishes department-wide standards, while delegating implementation to individual service branches. At the department level, DoD Instruction (DoDI) 1010.04, *Problematic Substance Use and Gambling Disorder*, serves as the overarching instruction, establishing procedures for the prevention, identification, diagnosis, and treatment of problematic alcohol use among military and civilian personnel [64]. Within the department-wide framework, each service branch operates its own alcohol and substance use program: the Army Substance Abuse Program [65], the Air Force Alcohol and Drug Abuse Prevention and Treatment Program, which also applies to the Space Force [66], the Navy Alcohol and Drug Misuse Prevention and Control Program [67], and the Marine Corps Substance Abuse Program [68]. All must conform to the standards set in DoDI 1010.04, and increasingly emphasize a patient-centered approach that integrates behavioral and pharmacological therapies for AUD [69]. Notably, while illicit drug use is addressed through a parallel deterrence-based framework centered on random urinalysis testing (DoDI 1010.01), no comparable biochemical screening program exists for alcohol [70]. Thus, identification of problematic drinking depends largely on self-report, clinical observation, command referral, or post-incident detection [71], all of which are vulnerable to underreporting and stigma-related suppression.

### Screening and Early Identification

There is a well-documented gap in identification of problematic drinking. Although post-deployment health assessments include validated alcohol screening items Alcohol Use Disorders Identification Test - Consumption (AUDIT-C), in a large cross-sectional analysis of Army active-duty members returning from Iraq or Afghanistan, nearly 29% screened positive for at-risk drinking on the AUDIT-C, yet interviewing providers identified potential alcohol problems in only 62% of those screening positive and referred only 29% for follow-up ^71^. Efforts to implement Screening, Brief Intervention, and Referral to Treatment (SBIRT) in military healthcare settings have shown promise but face military-specific challenges. A formative study in the Navy found broad support for the model but significant concerns about confidentiality and the truthfulness of self-report [72], and a randomized trial of SBIRT among military emergency department patients found some evidence of effectiveness among higher-risk participants but null results in intent-to-treat analyses [73].

### Prevention Strategies

There is a dearth of evidence for rigorously evaluated military-specific prevention programs. A recent systematic review identified only six evidence-based prevention programs with strong potential for military implementation [74], and a systematic review of workplace interventions for active-duty personnel found that while most showed short-term reductions in drinking, effects were not sustained and methodological rigor was generally low [75]. The most thoroughly evaluated prevention program is the Air Force Alcohol Misconduct Prevention Program, which combines a group-based brief alcohol intervention with random breathalyzer testing. Initial evaluation at Lackland Air Force Base showed a 45% reduction in alcohol-related incidents [76], and broad dissemination across four training bases reduced incidents by 16% [77].

Given the profound influence of leadership on individual behavior and organizational culture, the U.S. military has developed numerous avenues for educating leaders on alcohol and substance use prevention and intervention. Among these are embedded behavioral health professionals who serve as direct resources for commanders. In the U.S. Army, for example, brigade-level units are typically supported by behavioral health officers, including psychologists and clinical social workers, while division-level commanders have access to assigned psychiatrists who provide consultation, prevention, and clinical oversight across the formation [78–81]. These behavioral health assets have a well-established role in advising commanders on substance use, mental health readiness, and risk reduction across the deployment cycle. However, there are wide variations in implementation, both across and within services, as well as across installations, making it difficult to evaluate effectiveness for existing programs, processes, and policies.

### Treatment and Clinical Care

Clinical treatment for AUD within military health systems draws on the same evidence-based psychotherapies used in civilian settings, delivered in both individual and group formats as determined by clinical presentation and patient preference. Cognitive Behavioral Therapy (CBT) and Motivational Interviewing (MI) are the two most commonly employed modalities [82]. MI has been adapted specifically for the military context, including delivery via telephone to reach Service Members who avoid formal treatment due to confidentiality concerns. A randomized controlled trialT of telephone-delivered MI with Army soldiers with AUD found that MI participants reported significantly fewer drinks per week than controls at six months [83]. The military has also invested in training its clinical workforce in MI, with a randomized trial of MI training for Air Force behavioral health providers showing that structured training improved clinician skill levels, though gains required ongoing reinforcement to be sustained [84].

Beyond individual and group psychotherapy, mutual aid and peer recovery support services are available to Service Members and veterans using both in-person and virtual formats. However, on-installation delivery of these programs is not standard military practice. Formal peer support programs, including ones designed specifically for veterans and active-duty Service Members, have been developed within Veterans Affairs (VA) and in community organizations, and virtual delivery has expanded geographic reach, particularly for rural and deployed populations [85].

Although there are a variety of free-of-cost treatment and clinical care options available for active-duty Service Members through TRICARE and service branch programs, military alcohol and substance use treatment has faced significant criticism. A review by the Institute of Medicine characterized the system as being “decades behind the times”, noting an overreliance on inpatient care, minimal use of pharmacotherapy, and inadequate clinical staffing [86, 87]. The National Defense Authorization Act (NDAA) 2020, provisioned by the US Congress, specifically outlined requirements for the DoW to ensure care access for mental health, including alcohol and substance use disorders, including access to Medication Assisted Therapy (MAT) [88]. Despite this provision, pharmacotherapy for AUD remains substantially underutilized. Among active-duty Service Members with moderate-to-severe AUD, only 16% received any medication in 2017, up from 9% in 2010 [89].

FDA-approved pharmacotherapies for AUD, including naltrexone, disulfiram, and acamprosate, are available to Service Members, along with evidence-based options such as topiramate and gabapentin may also be prescribed. The VA/DoW Clinical Practice Guideline for Substance Use Disorders recommends naltrexone and topiramate as first-line treatments [90, 91]. However, Service Members receiving certain medications may be subject to deployment limitations, as many such medications present sustainment challenges in austere operational environments [92]. For some, sustained pharmacotherapy may ultimately require separation from service given its implications for readiness and worldwide deployability.

## Barriers to Treatment: Stigma, Career Consequences, and Confidentiality

Stigma and fear of career consequences remain the most significant barriers to treatment-seeking. Approximately 44% of military personnel endorse concern that leadership would treat them differently if they sought mental health care, and career worry is the strongest predictor of reluctance to seek treatment [93, 94]. This concern is not unfounded, as a systematic review found that those who sought treatment were more likely to be discharged, though the correlational design cannot establish causality [95]. The Army’s Confidential Alcohol Treatment and Education Pilot (CATEP), tested beginning in 2009, demonstrated that Service Members do use confidential treatment when given the opportunity [86], but, for unknown reasons, the pilot was not expanded or widely implemented [96]. In 2023, the DoW introduced policies to reduce stigma and barriers to care by allowing self-referral for behavioral health services, including alcohol treatment, without command notification [97]. However, this confidentiality only applies to personnel who meet specific criteria for voluntary care [97]. Further efforts to reduce barriers to help-seeking include the Brandon Act, which requires that Service Members requesting a mental health evaluation receive a confidential referral, thereby bypassing the chain-of-command involvement that has historically deterred voluntary care-seeking for alcohol and other behavioral health concerns [98]. However, there are little to no systematic examinations of the impact of such policies.

## Special Considerations

### Alcohol use Among Women in the Military

Women currently comprise approximately 21–22% of U.S. active-duty forces, reflecting an upward trend in representation across the military [99]. Alcohol-related risk profiles for women differ significantly from those of men in important and clinically meaningful ways, a disparity further complicated by the unique cultural and experiential context of military service.

Although prevalence data indicate that women drink less heavily than men, the gap is narrowing and may be misleading with respect to harm. Despite higher reported levels of binge, heavy, and hazardous drinking in active-duty men [100], women have shown equal or higher rates of dependence symptoms and productivity loss, and appear to be at risk for alcohol problems at lower levels of consumption [101]. In active-duty women, significant predictors of higher AUDIT scores, include drinking to cheer up, drinking to forget problems, anger propensity, and depressed mood; a pattern consistent with affect regulation and coping motives rather than the heavy social drinking that characterizes male consumption [102]. Earlier surveys in Army personnel found that, although men were more likely to engage in binge drinking, women exceeded safe consumption guidelines at a risk-adjusted rate nearly twice that of men, and women initially experienced greater psychosocial impairment at lower levels of consumption [103]. A large cross-sectional study found that new-onset AUD was diagnosed most frequently in active-duty men, suggesting that men carry the higher diagnostic burden [104]. However, there is a notable absence of detailed literature examining contributions of sex differences in alcohol-related risk profiles for military men and women.

### Alcohol Use Among Military Aviation Populations

Military aviators experience the same cultural exposure to heavy drinking as other Service Members, but operate in an environment with elevated safety and professional risk, where the consequences of alcohol disclosure can powerfully shape help-seeking behavior. In military aviation, alcohol-related diagnoses can result in immediate suspension of flying duties [105, 106] and mandatory rehabilitation [107]. Although therapy often permits the aviator to continue flying duties without adverse career impact, alcohol-related diagnoses can lead to, and are the most frequently occurring reason for, permanent disqualification [105–107]. Appropriate treatment often permits military aviators to return-to-flight status without adverse career impact 107, but the potential for higher severity in career consequences associated with receiving an alcohol-related diagnosis may contribute to underreporting and underdiagnosing in the military aviation population, creating an additional distinct barrier to treatment-seeking and care for aviators.

Research on alcohol issues among military aviators is scarcer than in ground forces, even though alcohol-related disorders are the most prevalent, yet under-reported, psychiatric conditions in the military aviation population [108]. Furthermore, evaluation of standard screens like AUDIT and Self-Administered Alcoholism Screening Test (SAAST) has shown that these screeners are less sensitive in aviators [108], increasing the risk of undetected alcohol problems. The Common Alcohol Logistical Scale-Revised (CAL-R) has demonstrated the best sensitivity and specificity among instruments tested in aviators, but its use requires a psychologist and its availability is limited [108]. Another study assessing AUDIT-C performance among Air Force Medical service personnel post-deployment found overall low sensitivity for AUD diagnosis [109], underscoring that screening tools validated in ground force populations do not automatically perform well in aviator populations.

## Future Directions and Research Needs

Despite evidence that perceived drinking norms, unit culture, and structural characteristics of the military environment impact alcohol use. Further research is required to deepen the understanding of the interplay of military culture, leadership attitudes, and the command environment. Additionally, how group and unit characteristics and identification factors contribute to alcohol use patterns, is not well understood and merits further investigation. This is of particular importance as the same social connections that can typically provide a buffer against psychological distress can also serve to drive and normalize heavy drinking behaviors in military populations. Understanding how the same group affiliations that promote psychological support may also contribute to stigma against acknowledging and addressing alcohol problems is essential for developing interventions and programs that work with military culture instead of against it.

Program evaluation is a substantial gap in this literature, highlighting the need for comprehensive and rigorous evaluation of the effectiveness of prevention and treatment programs that are implemented within the military. Many recent and current intervention and prevention strategies, while based on good population health principles, often lack robust outcome measures, largely relying on self-reported drinking without linking results to readiness, retention, or duty fitness. Future evaluations should adopt standardized, operationally relevant outcome metrics.

Finally, there is a critical need to expand the evidence base for populations underrepresented in the literature. Women and military aviators present distinct risk profiles and help-seeking contexts that are not adequately captured in studies of active-duty ground forces, and the existing evidence is insufficient to guide intervention design to fit the specific needs of these sub-populations. Dedicated prevalence studies for active-duty women remain sparce, and analyses of trends in alcohol use among Servicewomen have only recently emerged. Critically, no clinical trial evidence exists specifically evaluating alcohol interventions designed or adapted for active-duty women. For the aviator population, future research should develop and validate screening approaches that are both sensitive in the aviator population and compatible with the aeromedical disclosure environment. Additionally, further examination of whether the waiver and return-to-flight pathway functions as a meaningful treatment engagement mechanism is warranted.

## Conclusions

Alcohol misuse remains one of the most consequential and underaddressed behavioral health challenges facing the active-duty military. The evidence reviewed here makes clear that harmful drinking is not simply a personal health concern, it is structurally produced, culturally sustained, and operationally costly in ways that are well-documented and, to a meaningful degree, preventable. Despite substantial DoW investments in prevention, screening, treatment, and policy improvement, the prevalence of unhealthy drinking behaviors among military personnel remains consistently higher than in the civilian population, fewer than one in five Service Members with moderate-to-severe AUD receive pharmacotherapy, and high risk populations, including women, aviators, and combat-exposed personnel, remain underserved by an evidence base built largely around other groups. Closing this gap will require moving beyond programs that address alcohol use in isolation toward integrated approaches that account for the cultural context in which drinking occurs, the comorbidities that sustain it, and the institutional dynamics that deter help-seeking. It is essential for future efforts to evaluate outcomes that are operationally relevant, and provide clear, actionable data for optimizing readiness, retention, duty fitness, and force lethality.

## Key References

- Meadows SO, Beckman R, Engel CC, Jeffery DD. The Culture of Alcohol in the U.S. Military: Correlations With Problematic Drinking Behaviors and Negative Consequences of Alcohol Use. Armed Forces & Society. 2023;49(2):531-555. doi:10.1177/0095327X211069162
  - ○ The primary source for evidence linking perceived military drinking culture and harmful outcomes (excessive drinking, risky driving, productivity loss).
- Meadows SO, Engel CC, Collins RL, et al. 2018 Health Related Behaviors Survey: Substance Use Among the Active Component. 2021. Accessed March 27, 2026. https://www.rand.org/pubs/research_briefs/RB10116z3.html
  - ○ Highly relevant, foundational epidemiological report providing recent prevalence data for alcohol use in the active component of the U.S. military.
- Jacobson IG, Williams EC, Seelig AD, Littman AJ, Maynard CC, Bricker JB, Rull RP, Boyko EJ, Millennium Cohort Study Team (2020) Longitudinal Investigation of Military-specific Factors Associated With Continued Unhealthy Alcohol Use Among a Large US Military Cohort. J Addict Med 14:e53–e63
  - ○ The Millennium Cohort Study has tracked drinking behaviors prospectively across up to 12 years and linked alcohol misuse to separation status, service component, and combat exposure, providing foundational longitudinal infrastructure.
- Stahre MA, Brewer RD, Fonseca VP, Naimi TS. Binge drinking among U.S. active-duty military personnel. Am J Prev Med. 2009;36(3):208-217. doi:10.1016/j.amepre.2008.10.017
  - ○ Individual-level outcome data translating prevalence into operational outcomes.

## Data Availability

No datasets were generated or analysed during the current study.

## Declarations

### Disclaimers

The opinions and assertions expressed herein are those of the author(s) and do not reflect the official policy or position of the Henry M Jackson Foundation for the Advancement of Military Medicine, Inc., Uniformed Services University of the Health Sciences or the Department of War. References to non-Federal entities or products do not constitute or imply a Department of War or Uniformed Services University of the Health Sciences endorsement.

## References

[1] Le Daré B, Gicquel T. Therapeutic Applications of Ethanol: A Review. J Pharm Pharm Sci. 2019;22(1):525–35. 10.18433/jpps30572. 31604058 10.18433/jpps30572

[2] Rosso AM. Beer and wine in antiquity: beneficial remedy or punishment imposed by the Gods? Acta Med Hist Adriat. 2012;10(2):237–62. 23560753

[3] Whitby JD. Alcohol in anaesthesia and surgical resuscitation. Anaesthesia. 1980;35(5):502–5. 10.1111/j.1365-2044.1980.tb03830.x. 6994523 10.1111/j.1365-2044.1980.tb03830.x

[4] Houghton IT. A career in military anaesthesia. J R Army Med Corps. 2000;146(3):248–52. 10.1136/jramc-146-03-17. 11143697 10.1136/jramc-146-03-17

[5] Ames G, Cunradi C. Alcohol Use and Preventing Alcohol-Related Problems Among Young Adults in the Military. Alcohol Res Health. 2004;28:252–7.

[6] Kent ML, Upp JJ, Buckenmaier CC. Acute pain on and off the battlefield: what we do, what we know, and future directions. Int Anesthesiol Clin. 2011;49(3):10–32. 10.1097/AIA.0b013e318214d8f2. 21697667 10.1097/AIA.0b013e318214d8f2

[7] Houghton IT. Some observations on early military anaesthesia. Anaesth Intensive Care. 2006;34(Suppl 1):6–15. 10.1177/0310057X0603401S01. 16800222 10.1177/0310057X0603401S01

[8] Jones E, Fear NT. Alcohol use and misuse within the military: a review. Int Rev Psychiatry. 2011;23(2):166–72. 10.3109/09540261.2010.550868. 21521086 10.3109/09540261.2010.550868

[9] Meadows SO, Beckman R, Engel CC, Jeffery DD. The Culture of Alcohol in the U.S. Military: Correlations With Problematic Drinking Behaviors and Negative Consequences of Alcohol Use. Armed Forces Soc. 2023;49(2):531–55. 10.1177/0095327X211069162.

[10] Bray RM, Hourani LL. Substance use trends among active duty military personnel: findings from the United States Department of Defense Health Related Behavior Surveys, 1980–2005. Addiction. 2007;102(7):1092–101. 10.1111/j.1360-0443.2007.01841.x. 17567397 10.1111/j.1360-0443.2007.01841.x

[11] Bray RM, Pemberton MR, Lane ME, Hourani LL, Mattiko MJ, Babeu LA. Substance use and mental health trends among U.S. military active duty personnel: key findings from the 2008 DoD Health Behavior Survey. Mil Med. 2010;175(6):390–9. 10.7205/milmed-d-09-00132. 20572470 10.7205/milmed-d-09-00132

[12] Bray RM, Brown JM, Williams J. Trends in Binge and Heavy Drinking, Alcohol-Related Problems, and Combat Exposure in the U.S. Military. Subst Use Misuse. 2013;48(10):799–810. 10.3109/10826084.2013.796990. 23869454 10.3109/10826084.2013.796990

[13] McGuffin JJ, Riggs SA, Raiche EM, Romero DH. Military and Veteran help-seeking behaviors: Role of mental health stigma and leadership. Mil Psychol. 33(5):332–40. 10.1080/08995605.2021.1962181 10.1080/08995605.2021.1962181 PMC10013222 38536252

[14] Clement S, Schauman O, Graham T, et al. What is the impact of mental health-related stigma on help-seeking? A systematic review of quantitative and qualitative studies. Psychol Med. 2015;45(1):11–27. 10.1017/S0033291714000129. 24569086 10.1017/S0033291714000129

[15] Thomas JL, Bliese PD, Jex SM. Interpersonal Conflict and Organizational Commitment: Examining Two Levels of Supervisory Support as Multilevel Moderators. J Appl Soc Psychol. 2005;35(11):2375–98. 10.1111/j.1559-1816.2005.tb02107.x.

[16] Military Health System. Update: Diagnoses of Mental Health Disorders Among Active Component U.S. Armed Forces, 2019–2023. Published online 2024. https://www.health.mil/News/Articles/2024/12/01/MSMR-Mental-Health-Update-2024.

[17] Meadows SO, Engel CC, Collins RL et al. *2018 Health Related Behaviors Survey: Substance Use Among the Active Component*. 2021. Accessed March 27, 2026. https://www.rand.org/pubs/research_briefs/RB10116z3.html

[18] CDC. Facts About Excessive Drinking. Drink Less, Be Your Best. October 11. 2024. Accessed March 25, 2026. https://www.cdc.gov/drink-less-be-your-best/facts-about-excessive-drinking/index.html

[19] Jacobson IG, Ryan MAK, Hooper TI, et al. Alcohol use and alcohol-related problems before and after military combat deployment. JAMA. 2008;300(6):663–75. 10.1001/jama.300.6.663. 18698065 10.1001/jama.300.6.663 PMC2680184

[20] Jacobson IG, Williams EC, Seelig AD, et al. Longitudinal Investigation of Military-specific Factors Associated With Continued Unhealthy Alcohol Use Among a Large US Military Cohort. J Addict Med. 2020;14(4):e53–63. 10.1097/ADM.0000000000000596. 31821191 10.1097/ADM.0000000000000596 PMC7280069

[21] Abraham TH, Cheney AM, Curran GM, Drummond KL. Drinking as routine practice among re-integrating National Guard and Reservists from Arkansas. Qualitative Res Med Healthc. 2020;4(2):9001. 10.4081/qrmh.2020.9001.

[22] Ames GM, Cunradi CB, Moore RS, Stern P. Military culture and drinking behavior among U.S. Navy careerists. J Stud Alcohol Drugs. 2007;68(3):336–44. 10.15288/jsad.2007.68.336. 17446972 10.15288/jsad.2007.68.336

[23] Neighbors C, Walker D, Rodriguez L, et al. Normative Misperceptions of Alcohol Use Among Substance Abusing Army Personnel. Military Behav Health. 2014;2(2):203–9. 10.1080/21635781.2014.890883.

[24] Junkin E, Lau-Barraco C, Stamates A. Normative Perceptions of Peer Drinking Distinguish High-Intensity Drinkers from Other Drinking Groups. Subst Use Misuse. 2024;59:69–78. 37740503 10.1080/10826084.2023.2259463 PMC10841369

[25] Ames GM, Duke MR, Moore RS, Cunradi CB. The Impact of Occupational Culture on Drinking Behavior of Young Adults in the U.S. Navy. J Mixed Methods Res. 2009;3(2):129–50. 10.1177/1558689808328534.

[26] Ames GM, Spera C. Prevention in the military: early results on an environmental strategy. Alcohol Res Health. 2011;34(2):180–2. 22330217

[27] Moore RS, Ames GM, Cunradi CB. Physical and social availability of alcohol for young enlisted naval personnel in and around home port. Subst Abuse Treat Prev Policy. 2007;2:17. 10.1186/1747-597X-2-17. 17603908 10.1186/1747-597X-2-17 PMC1934352

[28] Department of Defense. *Armed Services Exchange Policy, DODI 1330.09*. 2005.

[29] Jowers K. What troops need to know about commissaries and exchanges in 2025. March 5, 2025. Accessed March 30, 2026. https://www.militarytimes.com/news/your-military/2025/03/05/what-troops-need-to-know-about-commissaries-and-exchanges-in-2025/

[30] Jowers K. No more late-night alcohol sales at AAFES stores, starting Jan. 1. Military Times. December 13, 2023. Accessed April 8, 2026. https://www.militarytimes.com/news/your-military/2023/12/13/no-more-late-night-alcohol-sales-at-aafes-stores-starting-jan-1/

[31] White M. No more late night beer runs — AAFES to end overnight alcohol sales in 2024. Task & Purpose. November 30, 2023. Accessed April 8, 2026. https://taskandpurpose.com/news/no-more-late-night-beer-runs-aafes-to-end-overnight-alcohol-sales-in-2024/

[32] Suicide Prevention and Response Independent Review Committee. Preventing Suicide in the U.S. Military: Recommendations from the Suicide Prevention and Response Independent Review Committee. U.S. Department of Defense; 2023. https://media.defense.gov/2023/Feb/24/2003167430/-1/-1/0/SPRIRC-FINAL-REPORT.PDF.

[33] Adler A, GcGurk D, Stetz M, Bliese P. Military Occupational Stressors in Garrison, Training, and Deployed Environments. Published online August. 2004;9:18.

[34] Fink DS, Keyes KM, Calabrese JR, et al. Deployment and Alcohol Use in a Military Cohort: Use of Combined Methods to Account for Exposure-Related Covariates and Heterogeneous Response to Exposure. Am J Epidemiol. 2017;186(4):411–9. 10.1093/aje/kww230. 28482012 10.1093/aje/kww230 PMC5860008

[35] Ramchand R, Miles J, Schell T, Jaycox L, Marshall G, Tanielian T. Prevalence and Correlates of Drinking Behaviors Among Previously Deployed Military and Matched Civilian Populations. Military Psychol. 2011;23:6–21. 10.1080/08995605.2011.534407 PMC4196271 25324594

[36] Campbell-Sills L, Ursano RJ, Kessler RC, et al. Prospective risk factors for post-deployment heavy drinking and alcohol or substance use disorder among US Army soldiers. Psychol Med. 2018;48(10):1624–33. 10.1017/S0033291717003105. 29039285 10.1017/S0033291717003105 PMC6620021

[37] Na P, Schnurr P, Pietrzak R. Mental health of U.S. combat veterans by war era: Results from the National health and Resilience in veterans study. J Psychiatr Res. 2023;158:36–40. 36565542 10.1016/j.jpsychires.2022.12.019 PMC11929138

[38] Wilk JE, Bliese PD, Kim PY, Thomas JL, McGurk D, Hoge CW. Relationship of combat experiences to alcohol misuse among U.S. soldiers returning from the Iraq war. Drug Alcohol Depend. 2010;108(1):115–21. 10.1016/j.drugalcdep.2009.12.003. 20060237 10.1016/j.drugalcdep.2009.12.003

[39] Larson MJ, Wooten NR, Adams RS, Merrick EL. Military Combat Deployments and Substance Use: Review and Future Directions. J Social Work Pract Addictions. 2012;12(1):6–27. 10.1080/1533256X.2012.647586. 10.1080/1533256X.2012.647586 PMC3321386 22496626

[40] Hawn SE, Cusack SE, Amstadter AB. A Systematic Review of the Self-Medication Hypothesis in the Context of Posttraumatic Stress Disorder and Comorbid Problematic Alcohol Use. J Trauma Stress. 2020;33(5):699–708. 10.1002/jts.22521. 32516487 10.1002/jts.22521 PMC7572615

[41] Schumm JA, Chard KM. Alcohol and stress in the military. Alcohol Res. 2012;34(4):401–7. 10.35946/arcr.v34.4.04. 23584106 10.35946/arcr.v34.4.04 PMC3860389

[42] Luciano MT, Acuff SF, Olin CC, et al. Posttraumatic stress disorder, drinking to cope, and harmful alcohol use: A multivariate meta-analysis of the self-medication hypothesis. J Psychopathol Clin Sci. 2022;131(5):447–56. 10.1037/abn0000764. 35587413 10.1037/abn0000764 PMC9233097

[43] Blakey SM, Tsai J, Elbogen EB. Drinking to Cope with Posttraumatic Stress: A Nationally Representative Study of Men with and without Military Combat Experience. J Dual Diagn. 2021;17(2):101–12. 10.1080/15504263.2021.1891360. 33730991 10.1080/15504263.2021.1891360

[44] Mohr C, McCabe C, Haverly S, Hammer L, Carlson K. Drinking Motives and Alcohol Use: The SERVe Study of U.S. Current and Former Service Members. J Stud Alcohol Drugs. 2018;79:79–87. 29227235 10.15288/jsad.2018.79.79 PMC5894860

[45] Mitchell MM, Gallaway MS, Millikan AM, Bell M. Interaction of combat exposure and unit cohesion in predicting suicide-related ideation among post-deployment soldiers. Suicide Life Threat Behav. 2012;42(5):486–94. 10.1111/j.1943-278X.2012.00106.x. 22934836 10.1111/j.1943-278X.2012.00106.x

[46] Griffith J. Cross (Unit)-Level Effects of Cohesion on Relationships of Suicide Thoughts to Combat Exposure, Postdeployment Stressors, and Postdeployment Social Support. Behav Med. 2015;41(3):98–106. 10.1080/08964289.2014.987719. 26332927 10.1080/08964289.2014.987719

[47] Anderson L, Campbell-Sills L, Ursano RJ, et al. Prospective associations of perceived unit cohesion with postdeployment mental health outcomes. Depress Anxiety. 2019;36(6):511–21. 10.1002/da.22884. 30694009 10.1002/da.22884 PMC7058190

[48] Campbell-Sills L, Flynn PJ, Choi KW, et al. Unit cohesion during deployment and post-deployment mental health: is cohesion an individual- or unit-level buffer for combat-exposed soldiers? Psychol Med. 2022;52(1):121–31. 10.1017/S0033291720001786. 32517825 10.1017/S0033291720001786 PMC9341401

[49] Griffith J. Cohesiveness in previously deployed Army National Guard units: Implications for postdeployment behavioral health. Psychol Serv. 2022;19(3):443–54. 10.1037/ser0000541. 33956479 10.1037/ser0000541

[50] Breslau J, Setodji CM, Vaughan CA. Is cohesion within military units associated with post-deployment behavioral and mental health outcomes? J Affect Disord. 2016;198:102–7. 10.1016/j.jad.2016.03.053. 27011365 10.1016/j.jad.2016.03.053

[51] Anderson Goodell EM, Johnson RM, Latkin CA, Homish DL, Homish GG. Risk and protective effects of social networks on alcohol use problems among Army Reserve and National Guard soldiers. Addict Behav. 2020;103:106244. 10.1016/j.addbeh.2019.106244. 31838442 10.1016/j.addbeh.2019.106244 PMC7045418

[52] Orr MG, Prescott MR, Cohen GH, et al. Potentially modifiable deployment characteristics and new-onset alcohol abuse or dependence in the US National Guard. Drug Alcohol Depend. 2014;142:325–32. 10.1016/j.drugalcdep.2014.07.005. 25064024 10.1016/j.drugalcdep.2014.07.005

[53] Avery ML, McDevitt-Murphy ME. Impact of Combat and Social Support on PTSD and Alcohol Consumption in OEF/OIF Veterans. Military Behav Health. 2014;2(2):217–23. 10.1080/21635781.2014.891433. 10.1080/21635781.2014.891433 PMC4111153 25071980

[54] Mattiko M, Olmsted K, Brown J, Bray R. Alcohol use and negative consequences among active duty military personnel. Addict Behav. 2011;36:608–14. 21376475 10.1016/j.addbeh.2011.01.023

[55] Stahre MA, Brewer RD, Fonseca VP, Naimi TS. Binge drinking among U.S. active-duty military personnel. Am J Prev Med. 2009;36(3):208–17. 10.1016/j.amepre.2008.10.017. 19215846 10.1016/j.amepre.2008.10.017

[56] Herberman Mash HB, Fullerton CS, Ng THH, Nock MK, Wynn GH, Ursano RJ. Alcohol Use and Reasons for Drinking as Risk Factors for Suicidal Behavior in the U.S. Army. Mil Med. 2016;181(8):811–20. 10.7205/MILMED-D-15-00122. 27483518 10.7205/MILMED-D-15-00122

[57] Hoge CW, Toboni HE, Messer SC, Bell N, Amoroso P, Orman DT. The occupational burden of mental disorders in the U.S. military: psychiatric hospitalizations, involuntary separations, and disability. Am J Psychiatry. 2005;162(3):585–91. 10.1176/appi.ajp.162.3.585. 15741477 10.1176/appi.ajp.162.3.585

[58] Porter B, Rodriguez LM, Woodall KA, Pflieger JC, Stander VA. Alcohol misuse and separation from military service: A dyadic perspective. Addict Behav. 2020;110:106512. 10.1016/j.addbeh.2020.106512. 32623237 10.1016/j.addbeh.2020.106512

[59] Gray JC, Larson MJ, Moresco N, et al. The association of engagement in substance use treatment with negative separation from the military among soldiers with post-deployment alcohol use disorder. Drug Alcohol Depend. 2021;221:108647. 10.1016/j.drugalcdep.2021.108647. 33647586 10.1016/j.drugalcdep.2021.108647 PMC8136466

[60] Self AR, Oetting AA, Clausen SS, Stahlman S. Alcohol-related emergency department visits, hospitalizations, and co-occurring injuries, active component, U.S. Armed Forces, 2009–2018. MSMR. 2020;27(7):7–14. 32726109

[61] Harwood HJ, Zhang Y, Dall TM, Olaiya ST, Fagan NK. Economic implications of reduced binge drinking among the military health system’s TRICARE Prime plan beneficiaries. Mil Med. 2009;174(7):728–36. 10.7205/milmed-d-03-9008. 19685845 10.7205/milmed-d-03-9008

[62] Fisher CA, Hoffman KJ, Austin-Lane J, Kao TC. The Relationship between Heavy Alcohol Use and Work Productivity Loss in Active Duty Military Personnel: A Secondary Analysis of the 1995 Department of Defense Worldwide Survey. Mil Med. 2000;165(5):355–61. 10.1093/milmed/165.5.355. 10826382

[63] Li T, Waters TM, Kaplan EK, et al. Economic Analyses of an Alcohol Misconduct Prevention Program in a Military Setting. Mil Med. 2017;182(1–2):e1562–7. 10.7205/MILMED-D-16-00098. 28051974 10.7205/MILMED-D-16-00098

[64] Department of Defense. *Problematic Substance Use and Gambling Disorder, DOD Instruction 1010.04*. 2025.

[65] Department of the Army. *AR 600–85: The Army Substance Abuse Program*. 2020.

[66] Department of the Air Force. DAFI 44–121: Alcohol and Drug Abuse Prevention and Treatment (ADAPT) Program. Published online 2018.

[67] Department of the Navy. OPNAVINST 5350.4E: Navy Alcohol and Drug Misuse Prevention and Control. Published online March 28, 2022.

[68] Commandant of the Marine Corps. MCO 5300.17A: Marine Corps Substance Abuse Program. Published online June 25, 2018.

[69] Schrader C, Lenton A, Gertonson P, Rahimi A. Redeveloping Substance Abuse Treatment for Military Personnel. Curr Psychiatry Rep. 2018;20(6):45. 10.1007/s11920-018-0911-1. 29779198 10.1007/s11920-018-0911-1

[70] Department of Defense. *Military Personnel Drug Abuse Testing Program, DOD Instruction 1010.01*. 2025.

[71] Larson MJ, Mohr BA, Adams RS, Wooten NR, Williams TV. Missed Opportunity for Alcohol Problem Prevention Among Army Active Duty Service Members Postdeployment. Am J Public Health. 2014;104(8):1402–12. 10.2105/AJPH.2014.301901. 24922163 10.2105/AJPH.2014.301901 PMC4103229

[72] Holt M, Reed M, Woodruff SI, DeMers G, Matteucci M, Hurtado SL. Adaptation of Screening, Brief Intervention, Referral to Treatment to Active Duty Military Personnel in an Emergency Department: Findings From a Formative Research Study. Accessed April 8, 2026. 10.7205/MILMED-D-16-00333 10.7205/MILMED-D-16-00333 28810975

[73] Reed MB, Woodruff SI, DeMers G, et al. Results of a Randomized Trial of Screening, Brief Intervention, and Referral to Treatment (SBIRT) to Reduce Alcohol Misuse Among Active-Duty Military Personnel. J Stud Alcohol Drugs. 2021;82(2):269–78. 10.15288/jsad.2021.82.269. 33823974 10.15288/jsad.2021.82.269 PMC8864620

[74] Piscitello J, Heyman RE, Smith Slep AM, Hogan JN. A Systematic Review of Evidence-Based Prevention Approaches for Alcohol Problems with Viability for Military Implementation. Mil Med. 2025;190(11–12):e2328–38. 10.1093/milmed/usaf182. 40643960 10.1093/milmed/usaf182 PMC13449883

[75] Watterson JR, Gabbe B, Rosenfeld JV, Ball H, Romero L, Dietze P. Workplace intervention programmes for decreasing alcohol use in military personnel: a systematic review. BMJ Mil Health. 2021;167(3):192–200. 10.1136/bmjmilitary-2020-001584. 33361438 10.1136/bmjmilitary-2020-001584

[76] Klesges RC, Talcott W, Ebbert JO, et al. Effect of the Alcohol Misconduct Prevention Program (AMPP) in air force technical training. Mil Med. 2013;178(4):445–51. 10.7205/MILMED-D-12-00400. 23707832 10.7205/MILMED-D-12-00400

[77] Talcott GW, McMurry T, Ebbert J, et al. Dissemination of a Universally Delivered Brief Alcohol Intervention in United States Air Force Technical Training. J Addict Med. 2021;15(4):318–24. 10.1097/ADM.0000000000000763. 33122547 10.1097/ADM.0000000000000763

[78] Warner CH, Appenzeller GN, Barry MJ, Morton A, Grieger T. The Evolving Role of the Division Psychiatrist. Mil Med. 2007;172(9):918–24. 10.7205/MILMED.172.9.918. 17937353 10.7205/milmed.172.9.918

[79] Warner CH, Breitbach JE, Appenzeller GN, Yates V, Grieger T, Webster WG. Division Mental Health in the New Brigade Combat Team Structure: Part II. Redeployment and Postdeployment. Mil Med. 2007;172(9):912–7. 10.7205/MILMED.172.9.912. 17937352 10.7205/milmed.172.9.912

[80] Hoyt T, Garnica G, Marsh D, Clark K, Desadier J, Brodniak S. Behavioral health trends throughout a 9-month brigade combat team deployment to Afghanistan. Psychol Serv. 2015;12(1):59–65. 10.1037/ser0000016. 25419917 10.1037/ser0000016

[81] Curley JM, Warner CH. Maximizing the Division Psychiatrist’s Garrison Prevention Role to Meet the U.S. Army’s 21st Century Readiness Expectations. Mil Med. 2019;184(5–6):e183–91. 10.1093/milmed/usz017. 30793212 10.1093/milmed/usz017

[82] Teeters JB, Lancaster CL, Brown DG, Back SE. Substance use disorders in military veterans: prevalence and treatment challenges. SAR. 2017;8:69–77. 10.2147/SAR.S116720. 10.2147/SAR.S116720 PMC5587184 28919834

[83] Walker DD, Walton TO, Neighbors C, et al. Randomized trial of motivational interviewing plus feedback for soldiers with untreated alcohol abuse. J Consult Clin Psychol. 2017;85(2):99–110. 10.1037/ccp0000148. 27736113 10.1037/ccp0000148

[84] Moyers TB, Manuel JK, Wilson PG, Hendrickson SML, Talcott W, Durand P. A Randomized Trial Investigating Training in Motivational Interviewing for Behavioral Health Providers. Behav Cogn Psychother. 2008;36(2):149–62. 10.1017/S1352465807004055.

[85] Jain S, McLean C, Rosen CS. Is There a Role for Peer Support Delivered Interventions in the Treatment of Veterans With Post-Traumatic Stress Disorder? Mil Med. 2012;177(5):481–3. 10.7205/MILMED-D-11-00401. 22645871 10.7205/milmed-d-11-00401

[86] Levin A, IOM Report Critical of Substance Abuse Care for Troops. Psychiatric News. 2012;47:b1–16. 10.1176/pn.47.20.psychnews_47_20_1-b.

[87] Kuehn BM. Treatment of Substance Abuse in Military Hampered by Old-fashioned Approach. JAMA. 2012;308(18):1845–6. 10.1001/jama.2012.13704. 23149987 10.1001/jama.2012.13704

[88] 116th Congress. NATIONAL DEFENSE AUTHORIZATION ACT FOR FISCAL YEAR. 2020, Public Law 116 – 92. Published online 2019.

[89] Peters ZJ, Kincaid MW, Greenberg JG, Quah RF, Curry JC. Rates of Prescription Orders for United States Active Duty Service Members Diagnosed with Alcohol use Disorder. Substance abuse. 2021;42(4):638–45. 10.1080/08897077.2020.1809604. 32870103 10.1080/08897077.2020.1809604

[90] Department of Veterans Affairs (VA) and U.S. Department of Defense (DoD). *VA/DoD Clinical Practice Guideline for the Management of Substance Use Disorders*. U.S. Department of Veterans Affairs and U.S. Department of Defense (VA/DoD); 2021. Accessed April 27, 2026. https://www.healthquality.va.gov/guidelines/mh/sud/

[91] Perry C, Liberto J, Milliken C, et al. The Management of Substance Use Disorders: Synopsis of the 2021 U.S. Department of Veterans Affairs and U.S. Department of Defense Clinical Practice Guideline. Ann Intern Med. 2022;175(5):720–31. 10.7326/M21-4011. 35313113 10.7326/M21-4011

[92] Costantino RC, Brandt NJ, Perfetto EM, Hull JR, Daniel Mullins C. Identifying Concepts to Establish Problematic Medications in Active Duty Servicemembers (ProMADS). Accessed April 27, 2026. 10.1093/milmed/usaf037 10.1093/milmed/usaf037 39954054

[93] Sharp ML, Fear NT, Rona RJ, et al. Stigma as a Barrier to Seeking Health Care Among Military Personnel With Mental Health Problems. Epidemiol Rev. 2015;37(1):144–62. 10.1093/epirev/mxu012. 25595168 10.1093/epirev/mxu012

[94] Brown NB, Bruce SE. Stigma, career worry, and mental illness symptomatology: Factors influencing treatment-seeking for Operation Enduring Freedom and Operation Iraqi Freedom soldiers and veterans. Psychol Trauma: Theory Res Pract Policy. 2016;8(3):276–83. 10.1037/tra0000082. 10.1037/tra0000082 26390109

[95] Heyman RE, Slep AMS, Parsons AM, Ellerbeck EL, McMillan KK. Systematic Review of the Military Career Impact of Mental Health Evaluation and Treatment. Accessed April 27, 2026. 10.1093/milmed/usab283 10.1093/milmed/usab283 34322709

[96] Gibbs DA, Rae Olmsted KL. Preliminary Examination of the Confidential Alcohol Treatment and Education Program. Military Psychol. 2011;23(1):97–111. 10.1080/08995605.2011.534418.

[97] Department of Defense. *DoD Instruction 6490.08: Command Notification Requirements to Dispel Stigmas in Providing Mental Health Care to Service Members*. 2023.

[98] Rep, Moulton. S [D M 6. H.R.3942–117th Congress (2021–2022): Brandon Act. June 16, 2021. Accessed April 27, 2026. https://www.congress.gov/bill/117th-congress/house-bill/3942

[99] Department of Defense. 2023 *Demographics Profile of the Military Community.* Office of the Deputy Assistant Secretary of Defense for Military Community and Family Policy; 2024.

[100] Meadows SO, Engel CC, Collins RL et al. *2015 Health Related Behaviors Survey: Summary Findings and Policy Implications*. 2018. Accessed April 28, 2026. https://www.rand.org/pubs/research_briefs/RB9955.html

[101] Brown JM, Bray RM, Hartzell MC. A Comparison of Alcohol Use and Related Problems Among Women and Men in the Military. Mil Med. 2010;175(2):101–7. 10.7205/MILMED-D-09-00080. 20180479 10.7205/milmed-d-09-00080

[102] Jeffery DD, Mattiko M. Alcohol Use Among Active Duty Women: Analysis AUDIT Scores From the 2011 Health-Related Behavior Survey of Active Duty Military Personnel. Mil Med. 2016;181(suppl1):99–108. 10.7205/MILMED-D-15-00222. 26741908 10.7205/MILMED-D-15-00222

[103] Lande RG, Marin BA, Chang AS, Lande GR. Gender differences and alcohol use in the US Army. J Am Osteopath Assoc. 2007;107(9):401–7. 17908832

[104] Judkins JL, Smith K, Moore BA, Morissette SB. Alcohol use Disorder in Active Duty Service Members: Incidence Rates over a 19-Year Period. Substance abuse. 2022;43(1):294–300. 10.1080/08897077.2021.1941512. 34214408 10.1080/08897077.2021.1941512

[105] Curry IP, Kelley AM, Gaydos SJ. Clinical Diagnoses Leading to Suspension in Army Aircrew: An Epidemiological Study. Published online July. 2018;1. 10.3357/AMHP.5048.2018. 10.3357/AMHP.5048.2018 29921349

[106] McCrary BF, Van Syoc DL. Permanent flying disqualifications of USAF pilots and navigators (1995–1999). Aviat Space Environ Med. 2002;73(11):1117–21. 12433238

[107] Franzos MA, Franzos TL, Woolford JS, McDonald WA. Alcohol abuse or dependence in the military aviator: guidance for the non-flight surgeon. Mil Med. 2012;177(10):1191–5. 10.7205/milmed-d-12-00026. 23113446 10.7205/milmed-d-12-00026

[108] Gates T, Duffy K, Moore J, Howell W, McDonald W. Alcohol screening instruments and psychiatric evaluation outcomes in military aviation personnel. Aviat Space Environ Med. 2007;78(1):48–51. 17225482

[109] Tvaryanas AP, Maupin GM, White ED, Schroeder VM, Mahaney HJ. The Performance of the AUDIT-C and the Examination of Risks Associated With Postdeployment Alcohol Misuse in Air Force Medical Service Personnel. Military Psychol. 2017;29(4):327–35. 10.1037/mil0000167.

